# Diagnostic Accuracy and Usability of the Pluslife *Mycobacterium tuberculosis* Assay on Tongue and Sputum Swabs in Symptomatic and Asymptomatic Adults in South Africa

**DOI:** 10.3390/diagnostics16172760

**Published:** 2026-08-28

**Authors:** Anura David, Lesley E. Scott, Lyndel Singh, Puleng Marokane, Manuel P. da Silva, Danielle Gast, Lara D. Noble, Ziyaad Waja, Tumelo Moloantoa, Neil A. Martinson, Wendy Stevens

**Affiliations:** 1 Wits Diagnostics Innovation Hub, Health Sciences Research Office, Faculty of Health Sciences, University of the Witwatersrand, Johannesburg 2193, South Africa; lesley.scott@wits.ac.za (L.E.S.); lara.noble@wits.ac.za (L.D.N.); wendy.stevens@wits.ac.za (W.S.); 2 Wits Diagnostics Innovation Hub, Wits Health Consortium (PTY) Ltd., Johannesburg 2193, South Africa; lyndel.singh@witsdih.ac.za (L.S.); danielle.gast@witsdih.ac.za (D.G.); 3 National Priority Programmes, National Health Laboratory Services, Johannesburg 2193, South Africa; puleng.marokane@nhls.ac.za (P.M.); drarri@hotmail.com (M.P.d.S.); 4 Perinatal HIV Research Unit (PHRU), University of the Witwatersrand, Johannesburg 1862, South Africa; wajaz@phru.co.za (Z.W.); moloantoat@phru.co.za (T.M.); martinson@phru.co.za (N.A.M.); 5 Center for TB Research, Johns Hopkins University, Baltimore, MD 21218, USA

**Keywords:** *Mycobacterium tuberculosis* complex, near point-of-care, tongue swabs, sputum swabs, Pluslife MTB

## Abstract

**Background/Objectives**: Access to accurate tuberculosis (TB) diagnostics remains limited, particularly in high-burden settings. The Pluslife MTB assay is among the first molecular tests specifically designed for swab-based detection of *Mycobacterium tuberculosis* complex (MTBC) and offers near point-of-care use. **Methods**: We conducted a prospective diagnostic accuracy study in South Africa to evaluate the performance of the Pluslife assay on tongue swabs (TSs) and sputum swabs (SSs) among symptomatic and asymptomatic adults. Results were compared against liquid culture as the reference standard and Xpert MTB/RIF Ultra (Xpert Ultra) as a comparator. Operational characteristics and ease-of-use were assessed through structured observation and Likert-scale scoring by testing personnel. **Results**: Of the 256 participants enrolled, 92 [36%] were people with HIV (PHIV) and 217 were included in the final analysis. Culture confirmed TB in 41/217 (19%). Pluslife on SSs demonstrated a sensitivity of 85% (95% CI: 70.8–94.4), compared to 83% (95% CI: 67.9–92.8), for Xpert Ultra on sputum and detected one additional case (paired analysis, *p* = 1.000). Sensitivity on TSs was lower (63%, 95% CI: 46.9–77.9), particularly among PHIV. Specificity exceeded 97% across specimen types. Concordance on TSs between Pluslife and Xpert Ultra increased with higher bacterial loads. Operational evaluation showed short hands-on time and high ease-of-use scores, though limitations were noted for patient identifier recording and troubleshooting on the Pluslife MiniDock device. **Conclusions**: The Pluslife MTB assay on SSs demonstrated good diagnostic accuracy and favorable usability in this study. Tongue swabs remain feasible but less reliable, supporting sputum as the preferred first specimen for TB diagnosis.

## 1. Introduction

Of the 8.3 million people who were reported as newly diagnosed with TB in 2024, only 54% were initially tested with a WHO-recommended rapid test (WRD) [1]. Barriers to the uptake of available technologies include factors such as high costs, supply chain issues, lack of local technical support, requirements for equipment maintenance, human resource and training gaps and health system and infrastructure constraints [2,3]. Therefore, improving access to accurate diagnostics remains essential for strengthening TB control. The growing diagnostic pipeline, along with the WHO’s shift to endorsing classes of diagnostics, offers countries increased flexibility to tailor testing approaches to both centralized and decentralized service-delivery models [4,5] for TB. The most recent definitions now distinguish between automated and manual low-complexity nucleic acid amplification tests (LC-NAATs) [2]. This shift is intended to enable TB testing closer to the patient at a reduced cost. The latest Tuberculosis Diagnostics Pipeline report [6] from the Treatment Action Group also highlights the emergence of near point-of-care (NPOC) technologies and the need for non-sputum specimens. Oral swabs, particularly tongue swabs (TSs), are a more accessible specimen that may improve case detection for individuals who are unable to produce sputum [7] and have recently been recommended by the WHO for TB diagnosis [8]. Most swab evaluation studies thus far have paired downstream testing of swabs on existing technologies designed for sputum [9,10] or on in-house polymerase chain reaction (PCR) assays [11].

The Pluslife MTB assay (Pluslife Biotech, Guangzhou, China) is a NPOC nucleic acid amplification test (NAAT) for the detection of *Mycobacterium tuberculosis* complex (MTBC) DNA, targeting the multicopy insertion sequence IS*6110* and the *gyrB* gene. The assay uses Pluslife’s proprietary RNase Hybridization-Assisted Amplification (RHAM) technology, which is an isothermal nucleic acid amplification method and provides results in approximately 30 min. It is a novel assay designed to expand access to NAAT-based TB diagnosis in decentralized and resource-limited settings. It is also among the first molecular tests specifically developed for swab-based MTBC detection and can analyze both TSs and sputum swabs (SSs). Preliminary studies, conducted in both laboratory-based and community settings, primarily among populations with a HIV prevalence of ~20% [12,13], have reported sensitivities of 74–86% for TSs and 86–94% for SSs, with performance varying by symptom status and among people with HIV (PHIV) [14]. However, comprehensive evaluations of the assay’s diagnostic performance, operational characteristics, and usability across diverse populations remain limited.

This study aimed to evaluate the diagnostic accuracy of the Pluslife MTB assay on TSs and SSs in both symptomatic and asymptomatic adults in Johannesburg, South Africa. Additionally, we assessed the operational characteristics and usability of the assay, including workflow efficiency, ease-of-use, and training requirements, to inform its potential integration into routine clinical and screening settings.

## 2. Materials and Methods

### 2.1. Study Design

This cross-sectional, prospective diagnostic accuracy study evaluated the performance of the Pluslife MTB assay for the detection of MTBC on TSs and SSs. The study was reported according to the Standards for Reporting Diagnostic Accuracy Studies (STARD) guideline. A completed STARD checklist is provided as Supplementary Material. Results were compared with a Mycobacterial Growth Indicator Tube (MGIT) liquid culture reference (Becton, Dickinson and Company, Sparks, MD, USA) and with Xpert MTB/RIF Ultra (Xpert Ultra; Cepheid, Sunnyvale, CA, USA) as a comparator, both performed on sputum.

Assay ease-of-use was assessed using a Likert scale completed by testing personnel. A five-point scale was used to express the level of agreement with each aspect evaluated: 1 (very difficult), 2 (difficult), 3 (neutral), 4 (easy), and 5 (very easy); higher scores indicated more favorable ease-of-use characteristics. At least two operators completed the assessment, and an average score was assigned for each characteristic, followed by a category and overall ease-of-use score.

Operational characteristics of the Pluslife MTB assay and associated instrumentation were evaluated by four laboratory personnel, including one medical technologist and three scientists with 3–14 years of laboratory experience. All operators received one day of training from Pluslife covering assay procedures and instrument maintenance and operation. Usability was assessed using a standardized in-house evaluation tool, examining hands-on time, workflow complexity, ease of use, result interpretation, connectivity, training requirements, instrument performance, waste management, maintenance, and storage and shipping requirements. Data were collected through direct workflow observation, instrument assessment, and structured feedback from operators who were blinded to participants’ clinical information.

### 2.2. Participant Recruitment and Study Procedures

Adults were consecutively enrolled from 7 to 29 July 2025 at primary healthcare facilities in Soweto and Matlosana Municipality, North West Province. Eligible participants were ambulatory adults aged ≥ 18 years who were symptomatic or eligible for targeted universal testing for TB (TUTT) [15] and undergoing TB assessment. Study staff approached potentially eligible individuals attending the participating clinics, assessed eligibility, and invited them to enroll in the study.

**Figure 1:**
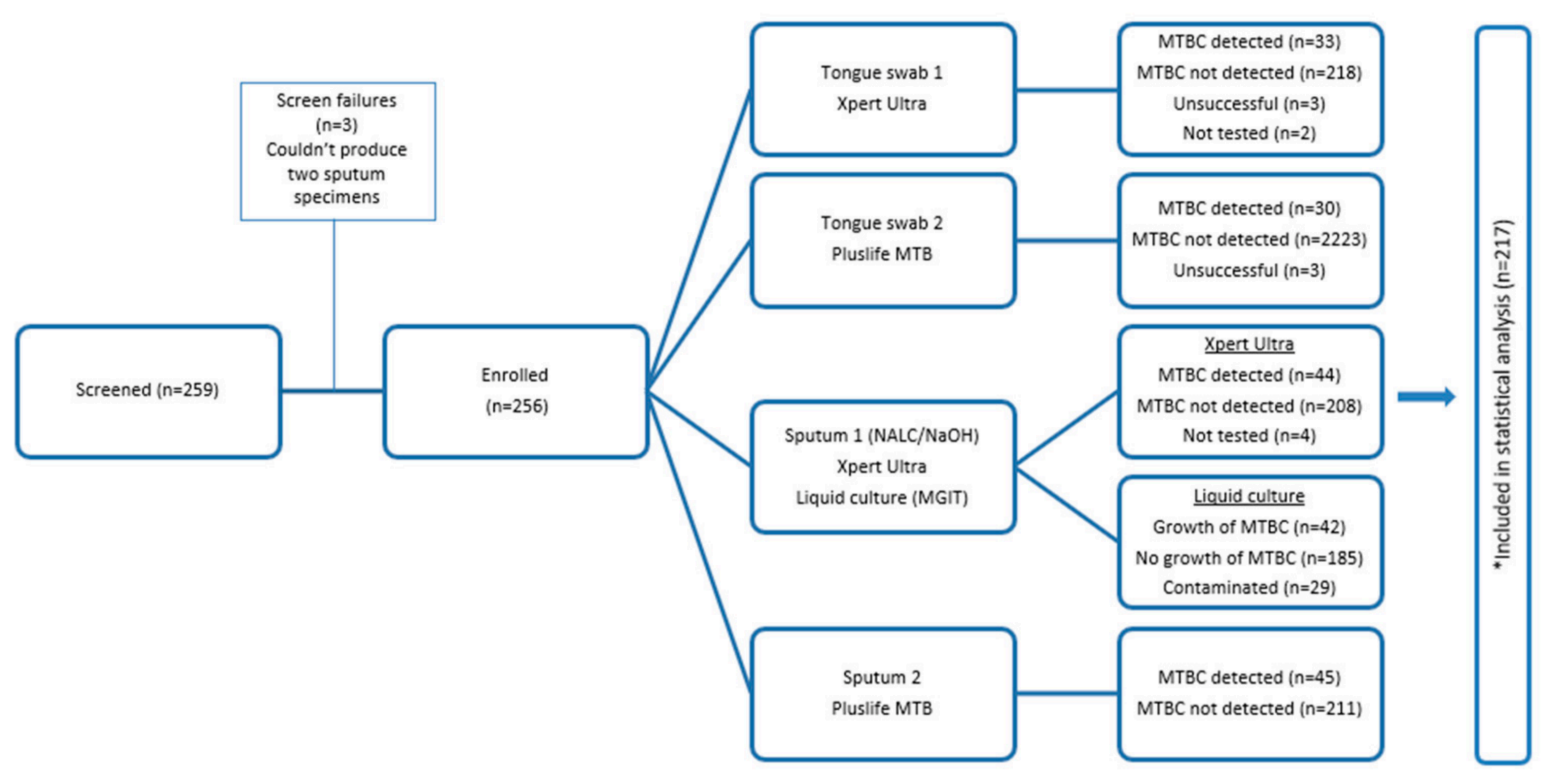
Specimen collection and diagnostic testing workflow at Visit 1. Participants first provided two tongue swabs followed by two sputum specimens. Tongue swabs were tested using Xpert Ultra and Pluslife MTB assays, while sputum specimens underwent Xpert Ultra, liquid culture (Mycobacterial Growth Indicator Tube [MGIT]), and Pluslife MTB testing. MTBC, *Mycobacterium tuberculosis* complex, * some participants had more than one unsuccessful test result.

The WHO-recommended four-symptom screen (cough, fever, weight loss, and night sweats) was administered to all participants. Participant characteristics including HIV status, TB history, diabetes mellitus status, medical conditions and demographic information were also collected. Two TSs and two sputum specimens were collected during a single study visit (Figure 1). The TSs were collected using the procedure described by Andama et al. [16], before sputum collection, with participants refraining from oral intake for at least 30 min prior. Briefly, TS collection from each participant was performed by research nurses, who swabbed the dorsum of the tongue with a Copan FLOQSwab with 80 mm breakpoint (Copan, Brescia, Italy) for 30 s, as far back as possible without initiating a gag reflex. The two sputum specimens were collected after three cycles of three forced-cough efforts unless a mucoid specimen was produced after the first effort. Sputum specimens were randomly assigned to either routine testing on Xpert Ultra or research testing using SS and tested on the Pluslife MTB assay.

Specimens collected for routine and research testing were transported to the Wits Diagnostic Innovation Hub (WitsDIH) laboratory in Johannesburg.

### 2.3. Routine Testing

Routine testing and result return was performed by laboratory staff as per the current National TB Management Guidelines [17]. Firstly, sputum specimens were processed using N-acetyl-L-cysteine/Sodium hydroxide (NALC/NaOH) for MGIT liquid culture using adapted procedures from the MGIT procedure manual [18] and the BACTEC MGIT 960 System User’s Manual. The decontaminated sputum was then tested on Xpert Ultra according to manufacturer instructions while TSs were processed using the diluted SR protocol by Ahls et al. [19]. Staff performing routine testing were blinded to results from the Pluslife MTB assay.

### 2.4. Pluslife MTB Testing

Sputum swabs and TSs were tested in the laboratory, by research personnel, as per manufacturer instructions, within 36 h of collection. Specimens were stored at 2–8 °C while awaiting testing. Testing personnel were blinded to routine testing results. For SS preparation in a biosafety cabinet, a clean swab (provided in the kit) was swirled 10 times in the sputum for ~10 s to allow the swab to be “dipped” in the sputum before processing. For processing of both TSs and SSs, the swab was added to the tube containing the nucleic acid releasing agent, lysed for 5 min using the Thermolyse device (Pluslife Biotech, Guangzhou, China), followed by testing on either the PM001 (MiniDock; Pluslife Biotech, Guangzhou, China) or the PM008 analyzer. The PM001 device can test a single specimen at a time while the PM008 format allows testing of eight specimens at a time. Results are interpreted as Positive, Negative or Invalid. Any participant who tested positive on any assay was re-contacted. Those who were positive on liquid culture or Xpert Ultra were immediately referred, with a copy of their results, for initiation of TB treatment if it had not already begun. For participants who tested positive only on the Pluslife MTB assay, and who were not yet on TB treatment, a second confirmatory sputum specimen was obtained and sent for routine TB diagnostic services. If this confirmatory test was positive, the patient was referred for initiation of TB treatment. Participants with any other discordant or invalid result were managed as per the national TB management guidelines [17].

### 2.5. Outcomes and Statistical Analysis

For performance evaluation, SSs and TSs results from Pluslife MTB were compared with the gold-standard liquid culture for MTBC detection. Diagnostic accuracy measures, including sensitivity, specificity, positive predictive value (PPV), and negative predictive value (NPV), were calculated with 95% confidence intervals (CIs) using the Wilson score method. Pluslife MTB results were additionally compared with Xpert Ultra on sputum.

Data analysis included calculation of sensitivity, specificity, positive predictive value (PPV) and negative predictive value (NPV), with 95% confidence intervals (CIs) calculated using the Wilson score method. Pluslife MTB results were additionally compared with Xpert Ultra on sputum.

The target sample size for the study was 200 participants, estimated using the standard sample size formula:

$$n\;=\;\frac{Z^{2}Xp(1-p)}{x^{2}}$$

where *n* is the required sample size, *Z* is the standard normal deviate corresponding to the desired confidence level (1.96 for 95% confidence), *p* is the estimated population proportion, and *x* is the desired precision (expressed as a proportion). A sample size of 200 participants corresponded to an estimated margin of error of approximately 6.9% at the 95% confidence level, which was considered sufficient for the study objectives. To account for potential participant dropout, missing data, and invalid test results, the sample size was increased by 10%, resulting in a target enrolment of 220 participants.

The primary analyses were pre-specified and included estimation of the sensitivity, specificity, PPV, and NPV of the Pluslife MTB assay on both TSs and SSs compared with liquid culture. Only participants with valid results across all evaluated assays (Pluslife MTB, Xpert Ultra, and liquid culture) were included in the primary performance analyses. Additional subgroup analyses, when conducted, were considered exploratory. Statistical analyses were conducted in Stata version 16 (StataCorp, College Station, TX, USA).

## 3. Results

### 3.1. Study Population Characteristics

Of the 256 participants screened for enrolment over a 1-month period, the average age of participants was 38 years and 133/256 (52%) were male (Table 1). A total of 92/255 (36%) were PHIV, with one participant reporting an unknown HIV status. Previous TB was reported by 39/256 (15%) participants. Most recruited participants were symptomatic (215/256 [84%]). Cough was the most frequently reported symptom, experienced by 207/215 (96%) of participants, and hemoptysis was the least frequently reported, being experienced by 12/215 participants (6%). A total of 3/256 (1%) participants showed elevated random glucose levels. Overall, 39/256 (15%) specimens had at least one unsuccessful test result (on Xpert Ultra, Pluslife MTB and/or culture). Of those with unsuccessful cultures, MTBC was detected in four participants using a sputum and/or TS molecular test. Statistical performance analysis was conducted on test results from 217 participants. The demographic and clinical characteristics of participants included in the analyses were comparable to those of participants who were excluded.

### 3.2. Diagnostic Accuracy of the Pluslife MTB Assay

Per bacteriological classification using culture, 41/217 (19%) were diagnosed with active TB disease (Table 2). A total of 166/217 (76%) were symptomatic while 51/217 (24%) participants were asymptomatic. The Pluslife MTB assay on SSs demonstrated better sensitivity (85%) than on TSs (63%). Assay performance on SSs yielded a sensitivity of 85%, compared with 83% for Xpert Ultra on sputum. Pluslife MTB detected MTBC in one additional specimen (McNemar’s *p* = 1.000). On TS, Xpert Ultra was able to detect MTBC in three additional specimens compared to Pluslife MTB.

Table 3 summarizes the level of agreement between the Pluslife MTB assays on TSs and SSs and the Xpert Ultra semiquantitative results, showing how agreement varied across different bacterial loads.

For specimens that yielded false-negative results using SSs, the liquid culture time-to-detection ranged from 7.16 to 38.21 days (mean: 20.32 days).

### 3.3. Unsuccessful Test Outcomes for Xpert Ultra and Pluslife MTB

The Xpert Ultra and Pluslife MTB assays each produced errors or invalid results in 3/256 (1.2%) TS tests. No unsuccessful results were observed on sputum with either assay. Although sufficient lysed specimen volumes were available to allow repeat testing with the Pluslife MTB assay, these were not performed. Availability of a lysed specimen eliminates the need to repeat the lysis step; only re-testing on the PM001 or PM008 analyzer is required.

### 3.4. Ease of Use and Operational Characteristics of the Pluslife MTB Assay

Overall, testing personnel reported that the Pluslife MTB system was straightforward to use across all operational categories (Table 4).

Operational considerations of the Pluslife MTB assay and instrumentation are summarized in Table 5.

## 4. Discussion

This study evaluated the performance of the Pluslife MTB assay using both TSs and SSs in a high-TB-burden setting. Participants were largely symptomatic, and a substantial proportion were living with HIV (36%), reflecting a population in which rapid diagnostic tools are most urgently needed. The assay demonstrated good diagnostic accuracy on SSs and identified one additional TB case compared with Xpert Ultra on sputum. Other studies which evaluated TS performance on the Pluslife MTB assay showed an overall sensitivity of ≥76% [12,13,14], though up to 60% of participants in these populations had high bacterial burdens, as indicated by their Xpert Ultra semiquantitative results. These studies, along with evaluations of other molecular assays [10,20], consistently show that sensitivity on TS decreases, in specimens with low bacterial burden, when compared to sputum. In our study, where 50% of participants demonstrated low bacterial burden, overall sensitivity on TS was 63%, when compared to liquid culture—lower than previously reported [12,13,14]. Another notable difference was the higher proportion of PHIV (36% versus ~20% in other studies), which may have contributed to the reduced TS performance. These findings suggest that while TSs offer a non-invasive and easily collected alternative specimen and are feasible for testing, they may be less reliable for routine diagnostic use, especially in paucibacillary disease.

When stratified by HIV status, diagnostic accuracy was reduced among PHIV, consistent with previous findings [21], Although performance on SSs remained relatively robust, sensitivity was lower on TS. These findings reinforce the value of sputum-based testing, even in settings where sputum collection can be difficult. Nevertheless, TS may provide a pragmatic alternative for individuals who are unable to expectorate sputum, despite its lower sensitivity.

Concordance between Pluslife MTB and Xpert Ultra increased with higher bacterial loads, with 100% agreement observed for specimens reported as “high” or “medium” by Xpert Ultra. Reduced agreement, at “low” and “trace” semiquantitative ranges, is expected for molecular assays and again mirrors the likelihood of fewer DNA targets at these concentrations. Tongue swabs were disproportionately affected by this trend, suggesting that sampling modality rather than assay chemistry may be responsible for the reduced sensitivity in low-burden disease.

The Pluslife MTB assay showed a low (1.2%) rate of unsuccessful tests and sufficient volume of lysed specimen is available for repeat testing. Training needs were minimal, with operators quickly gaining proficiency in specimen preparation, instrument operation, and result interpretation. Both the PM001 and PM008 analyzers were straightforward to install and use, and routine maintenance tasks were easily completed. Although result viewing and interpretation were generally intuitive, some limitations were identified for PM001, particularly around recording identifiers and accessing or exporting results, and occasional troubleshooting difficulties were reported. Despite these platform-specific issues, overall usability scores reflected high operator satisfaction. Operational findings should, however, be interpreted in the context of the usability assessment having been conducted by only four operators at a single site.

Hands-on time was short, with a simple four-step workflow for specimen processing. Both instruments were easy to operate, required limited training, and provided automated result read-outs. The absence of device interfaces meant that patient identifiers needed to be recorded manually for the PM001 device; however, connectivity options were available on PM008. Waste management, routine maintenance, and storage needs were minimal, supporting feasibility for routine implementation in low-resource settings.

This study has several limitations. The overall sample size was relatively small, and the number of culture-confirmed TB cases was limited, reducing the precision of diagnostic accuracy estimates, particularly in HIV-stratified analyses. Participants were enrolled over a relatively short period, which may not fully capture temporal variation in patient characteristics, disease presentation, or test performance. The study was conducted at a single high-TB-burden setting, limiting the generalizability of the findings. The predominance of symptomatic participants may also have introduced spectrum bias, as diagnostic test performance can differ between individuals with more severe disease and those with milder or asymptomatic disease. In addition, participants with missing, contaminated, or unsuccessful reference or Pluslife MTB test results were excluded from the final performance analyses. Although no meaningful differences in demographic or clinical characteristics were observed between included and excluded participants, the exclusion of individuals may have introduced some degree of verification bias. Finally, although the findings provide important preliminary evidence regarding the performance and usability of the Pluslife MTB assay, larger multicenter studies including a greater number of TB-positive participants and a broader spectrum of disease presentation are needed to provide more precise estimates of diagnostic accuracy.

Overall, this laboratory evaluation supports the use of the Pluslife MTB assay on SSs as a viable alternative to existing rapid diagnostics. One disadvantage of the Pluslife MTB assay is that it does not provide resistance testing, which means that diagnostic algorithms need to be developed to address this gap. Implementation in decentralized settings will require attention to biosafety concerns, including the risk of sputum spills, aerosol release during SS preparation, and safe disposal of residual sputum following testing. While TSs may increase bacterial yield [22] and offer operational advantages, our findings confirm that sputum collection remains essential wherever possible to ensure diagnostic accuracy. Performance on TSs underscores the need for continued refinement of sampling strategies if this minimally invasive approach is to be widely adopted.

Despite these considerations, the Pluslife MTB assay offers several practical advantages, including operational simplicity, short hands-on time, and favorable usability. These characteristics make it particularly attractive for decentralized and resource-limited settings where laboratory infrastructure and skilled personnel may be limited.

## 5. Conclusions

In conclusion, the Pluslife MTB assay demonstrated good diagnostic performance on sputum specimens and favorable operational characteristics. Although tongue swabs provide a less invasive sampling option, their lower sensitivity, particularly in people with HIV and low-bacterial-burden disease, underscores the continued importance of sputum for accurate TB diagnosis. Future work should focus on addressing the absence of resistance testing, while also informing biosafety practices and guiding workflow adaptations for safe and effective implementation. Additional research is also needed to assess assay performance in pediatric populations and evaluate cost-effectiveness in routine care settings.

## Figures and Tables

**Table 1:** Demographic, clinical, and symptom characteristics of study participants.

Characteristic	All (*n* = 256)
Demographics	
Age, mean (range), years	38 (18–73)
Male sex, *n* (%)	133 (51.9)
HIV-related information	
People with HIV, *n* (%)	92 (36.0)
HIV-negative, *n* (%)	163 (63.9)
Unknown, *n* (%)	1 (0.4)
Receiving * ARV therapy, *n*/total (%)	84/88 (95.5)
Elevated Random glucose levels, *n* (%)	3 (1.1)
Co-morbidities	
Hypertension, *n* (%)	19 (7.4)
Diabetes, *n* (%)	8 (3.1)
TB History	
Previously diagnosed with TB, *n* (%)	39 (15.2)
WHO four-symptom screen at presentation	
Cough, *n*/total (%)	207/215 (96.2)
Nights sweats, *n*/total (%)	89/215 (41.4)
Fever, *n*/total (%)	80/215 (37.2)
Unexplained weight loss, *n*/total (%)	52/215 (24.2)
Other reported symptoms, *n*/total (%)	
Fatigue, *n*/total (%)	60/215 (28.0)
Loss of appetite, *n*/total (%)	55/215 (25.6)
Body pain, *n*/total (%)	10/215 (4.7)
Hemoptysis, *n*/total (%)	12/215 (5.6)

* antiretroviral.

**Table 2:** The performance of Xpert Ultra and Pluslife MTB assays on tongue and sputum swabs compared to liquid culture for MTBC detection.

Performance Characteristic	Xpert Ultra Result				Pluslife MTB Result			
	Sputum	*n*/N	TS	*n*/N	SS	*n*/N	TS	*n*/N
All (*n* = 217)								
Sensitivity % (95% CI)	82.9 (67.9–92.8)	34/41	70.7 (54.5–83.9)	29/41	85.4 (70.8–94.4)	35/41	63.4 (46.9–77.9)	26/41
Specificity % (95% CI)	96.6 (92.7–98.7)	170/176	99.4 (96.9–100)	175/176	97.2 (93.5–99.1)	171/176	98.9 (96.0–99.0)	174/176
PPV % (95% CI)	85.0 (70.2–94.3)	34/40	96.7 (82.8–99.9)	29/30	87.5 (73.2–95.8)	35/40	92.9 (76.5–99.1)	26/28
NPV % (95% CI)	96.0 (92.0–98.4)	170/177	93.6 (89.1–96.6)	175/187	96.6 (92.8–98.7)	171/177	92.1 (87.2–95.5)	174/189
Symptomatic participants only (*n* = 166)								
Sensitivity % (95% CI)	87.5 (71.0–96.5)	28/32	75.0 (56.6–88.5)	24/32	90.6 (75.0–98.0)	29/32	68.8 (50.0–83.9	22/32
Specificity % (95% CI)	97.0 (92.5–99.2)	130/134	100 (97.3–100)	134/134	97.8 (93.6–99.5)	131/134	98.5 (94.7–99.8)	132/134
PPV % (95% CI)	87.5 (71.0–96.5)	28/32	100 (85.8–100)	24/24	90.6 (75.0–98.8)	29/32	91.7 (73.0–99.0)	22/24
NPV % (95% CI)	97.0 (92.5–99.2)	130/134	94.3 (89.1–79.5)	134/142	97.8 (93.5–99.5)	131/134	93.0 (87.3–96.5)	132/142
PHIV, irrespective of symptoms (*n* = 67)								
Sensitivity % (95% CI)	75.0 (50.9–91.3)	15/20	55.0 (31.5–76.9)	11/20	80.0 (56.3–94.3)	16/20	40.0 (19.1–63.9)	8/20
Specificity % (95% CI)	93.6 (82.5–98.7)	44/47	100 (92.5–100)	47/47	95.7 (85.5–99.5)	45/47	97.9 (88.7–99.9)	46/47
PPV % (95% CI)	83.3 (77.8–96.6)	15/18	100 (71.5–100)	11/11	88.9 (65.3–98.6)	16/18	88.9 (51.8–99.7)	8/9
NPV % (95% CI)	89.8 (77.8–96.6)	44/49	83.9 (71.7–92.4)	47/56	91.8 (80.4–97.7)	45/49	79.3 (66.6–88.8)	46/58
HIV-negative participants, irrespective of symptoms (*n* = 127)								
Sensitivity % (95% CI)	88.2 (63.6–98.5)	15/17	82.4 (56.6–96.2)	14/17	88.2 (63.6–98.5)	15/17	88.2 (63.6–98.5)	15/17
Specificity % (95% CI)	99.1 (95.0–100)	109/110	99.1 (95.5–100)	109/110	98.2 (93.6–99.8)	108/110)	99.1 (95.0–100)	109/110
PPV % (95% CI)	93.8 (69.8–99.8)	15/16	93.3 (68.1–99.8)	14/15	88.2 (63.6–98.5)	15/17	93.8 (69.8–99.8)	15/16
NPV % (95% CI)	98.2 (93.6–99.8)	109/111	97.3 (92.4–99.4)	109/112	98.2 (93.6–99.8)	108/110	98.2 (93.6–99.8)	109/111

TS, tongue swab; SS, sputum swab; *n*/N, the number of concordant results (*n*) out of the total number of specimens in that category (N); CI, confidence intervals; PHIV, people with HIV; PPV, positive predictive value; NPV, negative predictive value.

**Table 3:** Comparison of Xpert Ultra semiquantitative results with Xpert Ultra tongue swab, Pluslife MTB TS and sputum swab.

Xpert Ultra Semiquantitative Result (Sputum)	Xpert Ultra TS, *n* (% Agreement)	Pluslife MTB TS Result, *n* (% Agreement)	Pluslife MTB SS Result, *n* (% Agreement)
High (*n* = 13)	12 (92)	13 (100)	13 (100)
Medium (*n* = 7)	7 (100)	6 (85.7)	7 (100)
Low (*n* = 13)	10 (77)	7 (53.8)	13 (100)
Very low (*n* = 1)	0 (0)	0 (0)	1 (100)
Trace (*n* = 6)	0 (0)	0 (0)	1 (17)

TS, tongue swab; SS, sputum swab; MTB, *Mycobacterium tuberculosis*; *n*, the number of concordant results for a particular test.

**Table 4:** Ease-of-use table as scored by testing personnel for both analyzer formats.

Category	Characteristic	PM001	PM008
Training	Training on the use and storage of reagents	4.8	4.8
	Training on the use of the consumables	5.0	5.0
	Training on the use of the software	4.8	4.5
	Ease of result interpretation	5.0	5.0
Reagent and specimen preparation	Understand the specimen preparation protocol	5.0	5.0
	Ease of specimen preparation	4.8	4.8
	Understand the kit instructions for use	5.0	5.0
Use of instruments and software	Ease of instrument verification	^#^ 5.0	^#^ 5.0
	Understand the PM instrument manual	^ǂ^ 5.0	^ǂ^ 4.7
	Understand the device software manual	^ǂ^ 5.0	^ǂ^ 4.7
	Ease of use of the graphic user-interface	n/a	5.0
	Ease of use of the instrument software	n/a	4.8
	Ease of entering patient identifiers	n/a	3.5
	Ease of instrument set-up for a specimen run	5.0	4.8
	Ease of performing a specimen run	5.0	5.0
	Ease of performing daily instrument maintenance	5.0	5.0
	Ease of performing weekly instrument maintenance	^ǂ^ 5.0	^ǂ^ 5.0
	Ease of disposal of solid waste	5.0	5.0
	Ease of disposal of liquid waste	4.5	4.5
Result interpretation	Ease of viewing the result	4.8	5.0
	Ease in interpreting the result	5.0	5.0
	Ease of exporting the result	^ǂ^ 3.7	^ǂ^ 4.7
	Ease of accessing and viewing previous results	^ǂ^ 3.3	5.0
Troubleshooting	Understand error codes or flags for run failures	n/a	* 3.0
	Ease of troubleshooting frequently occurring errors	n/a	* 3.0
	Training	19.5/20 (97.5)	19.3/20 (96.5)
	Reagent and specimen preparation	14.8/15 (98.7)	14.8/15 (98.7)
	Use of instruments and software	44.5/45 (98.9)	56.8/60 (94.7)
	Result interpretation	16.8/20 (84)	19.7/20 (98.5)
	Troubleshooting	n/a	6/10 (60)
	Total	95.6/100 (95.6)	116.6/125 (93.3)

A Likert scale was used to assess the user’s experience for each characteristic, scored as follows: 5, very easy; 4, easy, 3, neutral; 2, difficult; 1, very difficult; n/a, not applicable. * The assigned score is a score from a single operator. ^#^ The assigned score is an average score from two operators. ^ǂ^ The assigned score is an average score from three operators. All other assigned scores are an average score from four operators. Subtotals represent the sum of individual ease-of-use scores within each category, expressed both as raw numbers and as percentages of the maximum possible score. The overall total reflects the sum of all category scores relative to the maximum possible score for that analyzer format.

**Table 5:** Operational considerations of the Pluslife MTB assay and instrumentation.

Consideration	Comment
Total Hands-on Time and Number of Steps	Specimen processing for the MTB assay involves four steps: addition to the sample release buffer (20 s), thermal lysis (5 min), addition of the lysed material to the testing cartridge (30 s) and testing on PM001/PM008 (12–25 min). Specimen registration on the PM008 is a manual process; hence, the overall hands-on time was 2–3 min.
Instruments	Both the thermolyze instrument and PM001/PM008 devices have user-friendly instructions The PM001 lacks a user interface, and patient identifiers cannot be entered. The user must manually record patient identifiers, cartridge serial numbers and other relevant information.
Result interpretation	At the end of the test run, the following occurs: o The PM008 screen displays the test result with no interpretation required by the user. o The PM001 uses a, LED indicator to display the test result with no interpretation required by the user. The LED indicator light switches off once the test card is removed from the device so the user must record the result prior to this. The instrument can be connected to a laptop, tablet or PC but since patient identifiers are not captured, the user needs to link the cartridge serial number and result to the patient.
Connectivity	The PM008 has an onboard printer and results can be printed. The PM001 needs to be connected to a laptop, tablet or PC to view the results. Since patient identifiers cannot be captured on the device, these need to be manually recorded. Wi-Fi connectivity can be used on the PM008 device to share results.
Training Needs	Minimal technical training was required; proficiency was achieved after 2–3 runs for a medical technologist and junior medical scientist.
Instrument Failure Rate	There was one occasion where a power outage caused failed runs. Two modules on the PM008 device demonstrated invalid and false-negative results, which resolved on repeat on other modules. These modules were not used for the duration of the study, leaving 6/8 modules available for testing.
Waste disposal	The sample release tubes, residual sputum and swab shafts were discarded into biohazard waste boxes for incineration prior to disposal. Test cards were discarded into sharps containers. The foil seals, swab pouches and package inserts were discarded into normal waste. The kit cardboard boxes were sent for recycling,
Maintenance and Customer Support Needs	Both the thermolyze device and the PM00 devices require minimal maintenance (cleaning the external surfaces of the instruments and discarding used cartridges). Customer support is easily accessible and responsive.
Shipping and Storage	The Pluslife MTB kits are transported and stored at room temperature.

## Data Availability

The protocol and raw data supporting the conclusions of this article will be made available by the authors on request.

## Supplementary Materials

The following supporting information can be downloaded at: https://www.mdpi.com/article/10.3390/diagnostics16172760/s1, Supplementary Material: STARD-2015-Checklist.

## Abbreviations

The following abbreviations are used in this manuscript:

TB	Tuberculosis
MTBC	*Mycobacterium tuberculosis* complex
TSs	Tongue swabs
SSs	Sputum swabs
WRD	WHO-recommended rapid test
NAATs	Nucleic acid amplification tests
PCR	Polymerase chain reaction
PHIV	People with HIV
MGIT	Mycobacterial Growth Indicator Tube
Wits DIH	Wits Diagnostic Innovation Hub
NALC/NaOH	N-acetyl-L-cysteine/Sodium hydroxide
PPV	Positive predictive value
NPV	Negative predictive value
WHO	World Health Organisation
